# Influence of dietary *Bacillus coagulans* and/or *Bacillus licheniformis*-based probiotics on performance, gut health, gene expression, and litter quality of broiler chickens

**DOI:** 10.1007/s11250-023-03453-2

**Published:** 2023-01-14

**Authors:** Ebtihal M. M. Elleithy, Basma M. Bawish, Shaimaa Kamel, Elshaimaa Ismael, Dina W. Bashir, Dalia Hamza, Khaled Nasr El-din Fahmy

**Affiliations:** 1 Department of Cytology and Histology, Faculty of Veterinary Medicine, Cairo University, Giza, 12211 Egypt; 2 Department of Veterinary Hygiene and Management, Faculty of Veterinary Medicine, Cairo University, Giza, 12211 Egypt; 3 Department of Biochemistry and Molecular Biology, Faculty of Veterinary Medicine, Cairo University, Giza, 12211 Egypt; 4 Department of Zoonoses, Faculty of Veterinary Medicine, Cairo University, PO Box 12211, Giza, 12211 Egypt; 5 Department of Nutrition and Clinical Nutrition, Faculty of Veterinary Medicine, Cairo University, Giza, 12211 Egypt

**Keywords:** *Bacillus coagulans*, *Bacillus licheniformis*, Broiler, Clostridia, Growth performance, *NBN*, *TLR*

## Abstract

Probiotics are non-pathogenic microorganisms that are potentially important non-antibiotic alternatives. This study aimed to compare novel multi-strain and single-strain *Bacillus* probiotics and their respective influences on broiler chickens’ performance, gut health, litter quality, immune response, and *NBN* and *TLR* gene expression. A total of 1200 Arbor-Acres 1-day-old broiler chicks were randomly allocated into three treatments (T1 was a control, T2 was supplemented with a combined *Bacillus coagulans* (2 × 10^9^ cfu/g) and *Bacillus licheniformis* (8 × 10^9^ cfu/g) probiotic strains (0.2 kg/ton of feed), and T3 was supplemented with *Bacillus licheniformis* (3.2 × 10^9^ cfu/g) probiotic (0.5 kg/ton of feed) with eight replicas of each. Supplementing the broiler diet with either the single-strain (T3) or the multi-strain (T2) *Bacillus*-based probiotic raised the overall birds’ body weight, body weight gain, feed conversion ratio, and European production efficiency factor compared to the control (T1), with a significant enhancement achieved by the multi-strain *Bacillus* product (*P* = 0.005). T2 and T3 exhibited significantly improved cholesterol, Alanine aminotransferase, aspartate aminotransferase, lactate dehydrogenase, and alkaline phosphatase levels than the control (*P* ≤ 0.05). The transcript levels of both *NBN* and *TLR* genes were upregulated in the liver in the T2 and T3 groups. The T2 group experienced significant reductions in gut bacterial counts, especially for *Clostridia*, and recorded the lowest litter moisture and nitrogen. In conclusion, supplementing broiler diets with probiotics of multiple *Bacillus* strains increased production profitability by promoting bird growth, improving feed intake, enhancing gut mucosa and immune organs, and upregulating genes responsible for immunity. All these inhibit the overgrowth of enteric pathogens and sustain litter quality.

## Introduction

In the previous 50 years, poultry producers integrated antibiotics as growth promoters into broiler diets at sub-therapeutic doses to control the overgrowth of gastrointestinal disease-causing microorganisms, predominantly *Clostridium perfringens* and coccidiosis (Mehdi et al., 2018). However, the frequent use of antimicrobials was implicated in the progression of bacterial resistance, which is a public health problem. Hence, the use of antibiotics as growth promoters has been banned by the European Union (ESVAC, 2017). Accordingly, the poultry feed industry sought out sustainable non-antibiotic alternatives to maintain a high production performance while protecting the environment and human health. Additionally, consumers are showing a growing interest in organic poultry meat (Hafez and Shehata, 2021).

Probiotics are beneficial live bacteria when supplemented in broiler feed can promote growth, intestinal health, and bird immunity to infections (Gernat et al., 2021; Tellez-Isaias et al., 2021). In addition, probiotics could modulate lipid metabolism and reduce serum cholesterol levels, through bile salts’ deconjugation with bile salt hydrolase enzyme (Reis et al., 2017; Hussien et al., 2022). The *Bacillus* bacteria are considered among the best probiotics discovered because of their immense attributes of releasing antimicrobial substances effective on pathogenic microorganisms, as well as their capacity to survive in harsh environments (Hong et al., 2005). Previous research studies focused on some bacilli probiotic strains, such as *B. subtilis*, *B. coagulans*, *B. licheniformis*, *B. cereus*, and *B. polymyxa* (Amoah et al., 2021).

*Bacillus coagulans* and *Bacillus licheniformis* are Gram-positive, spore-formers bacteria, that are commonly used as commercial probiotics for poultry feed (Moeller et al., 2009; Cutting, 2011). Due to their protective protein coat and other mechanisms, they can resist environmental stressors during the pelleting process, storage, and handling (Upadhaya et al., 2019a, b; Bonos et al., 2021), as well as survive stomach acidity and pass to the intestine where it germinates and grows safely without encoding for enterotoxins (Endres et al., 2009; Xing et al., 2021). *B. coagulans* is a homofermentative strain that utilizes hexose efficiently and has been recently considered a novel and safe probiotic. *B. coagulans* produces L-lactic acid as the primary byproduct of glucose fermentation (approximately 97% of the fermented products) while producing acetate and succinate as minor products (Qin et al., 2009; Endres et al., 2009). *B. coagulans* maintain the intestinal mucosal barrier by improving the intestinal flora, promoting the regeneration of broilers’ intestinal epithelial cells, and enhancing innate immunity (Liu et al., 2022). *B. licheniformis* have been extensively used in the poultry industry as a growth promoter to improve bird growth. Additionally, *B. licheniformis* was used as a prebiotic that combat *Clostridia* intestinal colonization in commercial broiler production (Zhou et al., 2016). Dietary supplementation of broiler diet with *B. licheniformis* could facilitate nutrient degradation and absorption by their ability to produce protease, lipase, and amylase enzymes and therefore increase feed efficiency and improve growth (Rozs et al., 2001).

To date, there are few published studies conducted on broiler chickens to examine the combined dietary *Bacillus coagulans* and *Bacillus licheniformis* probiotic supplementation to broiler chickens on birds’ performance, gut health, and immune responses. Consequently, the present research aims to evaluate and compare the efficacies of two products of bacilli-based probiotics on broiler’s growth performance, carcass traits, blood biochemistry, gut health, genes expression, and cecal bacteria, in addition to litter chemical and microbial qualities. The first product was a novel multi-strain *Bacillus* probiotic composed of both *Bacillus coagulans* (DSM 32,016; 2 × 10^9^ cfu/g) and *Bacillus licheniformis* (DSM 33,806; 8 × 10^9^ cfu/g), while the second probiotic product was from the commercial market and consisted of a single-strain, *Bacillus licheniformis* (DSM 17,236; 3.2 × 10^9^ cfu/g).

## Materials and methods

### Ethics approval

The current experiment was accepted according to the Institutional Animal Care and Use Committee guidelines, Faculty of Veterinary Medicine, Cairo University, Egypt. (Ethical reference No: Vet CU28/04/2021/280).

### Bird husbandry

The experimental study was performed at the Animal and Poultry Research Center, Faculty of Veterinary Medicine, Cairo University, Giza, Egypt. A total of 1200 Arbor-Acres 1-day-old, unsexed broiler chicks were obtained from a commercial hatchery, weighed (average initial weight 44.5 gm), and randomly allocated to three treatments (groups) in a completely randomized design, each treatment containing 8 replicates (pens) with 50 birds/replicate (stocking density was 10 birds/m^2^). Birds of different replicates were housed in separate floor pens (2.9 × 1.7 m) with a 7–10 cm height of clean wood shavings litter in a naturally ventilated open house for 35 days. The environmental temperature in the first 3 days of life was 33 °C, and it gradually decreased by 2.8 °C each week until it reached 24 °C and remained constant for the remainder of the experiment. The relative humidity was maintained at about 55–60% throughout the entire period of the trial. Birds were kept under 24-h lighting for the first 3 days and then maintained under 23 L:1 D for the remainder of the study. The vaccination schedule was as follows: on day 6, the birds were vaccinated against the Newcastle disease virus (NDV-Hitchner B1) through eye dropping. On day 14, the birds got infectious bursal disease (IBD) and avian influenza (H5N1) vaccines via S/C injections (0.2 ml/bird) and on day 18, they received the NDV-Lasota vaccine through eye droppings.

### Experimental design

The birds were fed a corn-soya bean meal-based diet that was formulated to meet the nutrient requirements for broilers according to the Arbor-Acres Broiler Nutrition Supplement (Aviagen, 2009), while feed and water were provided ad libitum. The birds were fed a starter crumble diet (1–14 days), which was changed to a grower mash diet instead of normal grower pellet form (15–27 days), and finally finisher pelleted diet (28–35 days) (Table 1). The birds were equally and randomly divided into three treatment groups. The birds in the first treatment group, which was considered the control group (T1), were only fed the basal diet (Table 1). The birds in the second treatment group (T2) were fed a basal diet supplemented with a multi-strain probiotic formula of naturally occurring strains of lactic acid producer *Bacillus coagulans* (DSM 32,016; 2 × 10^9^ cfu/g) and pathogen inhibitory *Bacillus licheniformis* (DSM 33,806; 8 × 10^9^ cfu/g) provided on a calcium carbonate carrier at a rate of 0.2 kg/ton of feed (TechnoCare®, BIOCHEM Zusatzstoffe Handels-und Produktionsgesellschaft mbH, Lohne, Germany). The birds in the third treatment group (T3) were fed a basal diet supplemented with a commercial single-strain probiotic product from the commercial market based on a naturally occurring strain of *Bacillus licheniformis* (DSM 17,236; 3.2 × 10^9^ cfu/g) provided on a calcium carbonate carrier at a rate of 0.5 kg/ton of feed (GalliPro® Tect, Chr. Hansen Holding A/S, Hørsholm, Denmark). This strain was selected specifically for its ability to germinate and multiply fast in the gut and its ability to interact with other microorganisms.

**Table 1:** Compositions (physical ingredients and chemical analysis) of basal diets administered in each period

Items	Starter (0 to 14 days)	Grower (15 to 28 days)	Finisher (29 to 35 days)
Ingredients%			
Yellow corn	55.24	59.39	63.64
Soybean meal 46% CP	27.00	18.60	10.30
Full fat SBM	8.00	12.50	16.00
Corn-gluten meal 60%	6.00	6.00	6.50
Monocalcium phosphate	0.90	0.80	0.80
Limestone	1.60	1.50	1.50
NaCl	0.35	0.35	0.35
Sod bicarbonate	0.10	0.10	0.10
L-Lysine	0.25	0.25	0.30
DL-Methionine	0.15	0.10	0.10
Toxin binder	0.10	0.10	0.10
Quantum blue (Phytase)	0.01	0.01	0.01
Broiler premix^1^	0.30	0.30	0.30
Chemical analysis			
ME (Kcal/kg)	3001.05	3100.63	3200.24
Crude protein (%)	23.17	21.11	19.14
Crude fat (%)	3.96	4.87	5.60
Calcium (%)	1.00	0.94	0.93
P. Available (%)	0.50	0.45	0.42

^1^Vitamin and mineral mixture contained vitamin A (13,000,000 IU); vitamin D3 (6,000,000 IU); vitamin E (80,000 mg); vitamin K (4000 mg); vitamin B1 (5000 mg); vitamin B2 (9000 mg); vitamin B6 (5000 mg); vitamin B12 (35 mg); pantothenic acid (20,000 mg); nicotinic acid (70,000 mg); folic acid (2000 mg); biotin (250 mg); choline chloride (400,000 mg); manganese oxide (120,000 mg); zinc oxide (100,000 mg); copper sulphate (15,000 mg); calcium Iodide (1000 mg); ferrous sulphate (50,000 mg); selenium selenite (350 mg)

### Growth performance

Body weights were recorded at arrival and once every week until 35 days of age (20 birds/group). The weekly body weight gains (BWG) and feed intakes (FI) were determined by calculating the changes between the primary and final weight, and between the offered and remaining quantity of feed, respectively. The FI/week was then divided by the total number of birds in each replicate after adjusting for mortality to get the average weekly FI/bird. Feed conversion ratio (FCR) was measured by dividing the weekly FI by the weekly BWG considering the dead birds. The European Production Efficiency Factor (EPEF) was calculated (Marcu et al., 2013) as follows:

$$\mathrm{EPEF}=(\mathrm{livability}\times\mathrm{live}\;\mathrm{body}\;\mathrm{weight}\;(\mathrm{kg})/(\mathrm{age}\;\mathrm{in}\;\mathrm{days}\times\mathrm{FCR})\times100$$

Throughout the experiment, the daily mortality was documented for every replicate and the mortality rate was calculated.

### Carcass traits and immune organs’ relative weights

At the age of 35 days, eight birds from each treatment were randomly selected (close to the average weight), weighed, and slaughtered after 12 h of fasting for complete gut evacuation. After removing the head, neck, and legs, each bird was scalded, de-feathered, and eviscerated, and the carcass was weighed and expressed as a percentage of its live weight (carcass yield). The carcass was dissected, and the relative weights of the breast, drumstick, and thigh were measured. In addition, the weights of the gizzard, liver (without gall bladder), heart, spleen, and bursa of Fabricius were recorded and their percentages relative to the carcass weight (the relative organ weights) were calculated.

### Sampling of litter and intestinal content

Litter sampling was carried out at 10, 23, and 35 days of age in each group (8 samples/group). The top 7 cm of deep litter were scooped from three different locations of each replicate pen and placed in sterile plastic bags (Dumas et al., 2011; Lopes et al., 2013). Cloacal swabs and cecal contents (eight samples per group) were collected from birds on days 23 and 35, respectively. All samples were stored at 4 °C till further bacteriological examination.

### Litter physical and chemical examinations

The moisture content of litter was determined as previously reported by Dumas et al. (2011). Weighed litter (10 g) from each replicate was subjected to drying at 100 °C ± 5 °C for 24–48 h in a hot air oven. The moisture content percentage was determined by subtracting dry litter weight from the initial weight. Determination of the total nitrogen content of the litter was calculated as total Kjeldahl nitrogen (Jackson, 1973).

### Microbiological examinations of litter and intestinal content

For microbial examinations of each litter sample, 3 g of homogenized litter were diluted in tubes containing 27 ml of sterile saline solution (10^−1^ dilution), left for 30–60 min at room temperature, and frequently shaken to allow the litter to mix well with the diluent (Dumas et al., 2011; Lopes et al., 2013). For microbial examinations of cecal contents, 1 g was diluted and homogenized in tubes containing 9 ml sterile saline solution (10^−1^ dilution) (Esmaeilipour et al., 2012). The samples were subjected to tenfold serial dilution in tubes containing 9 ml aliquots of sterile saline solution. From the serially diluted tubes, 100 µl samples were spread onto Nutrient Agar and Reinforced Clostridial Agar (Oxoid Ltd, Basingstoke, Hants, UK) to enumerate the total aerobes, total anaerobes, and *Clostridia*, respectively. Plates were then put in incubation at 37 °C for 24–48 h, and tightly sealed anaerobic jars were used to achieve anaerobic conditions. Finally, the counts of bacterial colonies for each 1 g of litters and caecal contents were reported as mean 10 logarithm colony forming units (log_10_CFU).

### Confirmation and toxin typing of *Clostridium perfringens*

The genomic DNA of suspected *Clostridium perfringens* isolates was extracted by means of an extraction kit (QIAamp mini kit, Qiagen, Hilden, Germany). The multiplex PCR assay was employed to identify the presence of genes encoding alpha-toxin (cpa), beta-toxin (cpb), epsilon-toxin (etx), iota toxin (iap), in addition to enterotoxin gene (cpe). Primer sequences were published previously (Ghoneim and Hamza 2017).

The multiplex PCR assay was conducted per the method described by Ghoneim and Hamza (2017). The PCR reaction mixtures were analyzed by electrophoresis on a 1.5% (w/v) agarose gel in the presence of a 100 bp DNA ladder (Fermentas Life Science, USA).

### Blood biochemical analysis

On day 35, blood samples (eight samples/group) were randomly collected for separation of serum by centrifugation at 5000 rpm for 15 min, and then stored at − 20 °C for further analysis. The serum samples were analyzed spectrophotometrically (UV-2100 Spectrophotometer, USA) for total cholesterol (at 500 nm wavelength), triglycerides (505 nm), total protein (546 nm), albumin (578 nm), globulin (calculated from the equation of total protein–albumin), albumin globulin ratio (A/G), alanine transaminase (ALT) (340 nm), aspartate transaminase (AST) (340 nm), lactate dehydrogenase (LDH) (340 nm), alkaline phosphatase (ALP) (405 nm), and uric acid (546 nm) using reagent kits of Spectrum Diagnostics Egyptian Company for Biotechnology, Egypt.

### Evaluation of the ND vaccinal immune status

As recommended by the OIE (2022), the hemagglutination inhibition (HI) test was used for evaluating the vaccinal immune status of the birds (8 samples/group). Two-fold dilutions were performed for 25 µl volumes of serum samples in 99-V-bottomed microwell plastic plates and tested against 25 µl volumes of four hemagglutination units (4 HAU) of the ND virus commercial antigen (Lasota). Plates were incubated at room temperature for 20 min, then 25 µl aliquots of a 1% suspension of packed chicken RBCs were added to each well. Antibody titers were reported as mean log_2_HI titers.

### Histological examination

On day 35, the liver, intestine, and immune organs “spleen and bursa of Fabricius” (eight samples of each organ/group) were dissected from bird carcasses. Sections from the middle of the duodenum, jejunum, ileum (all measuring about 0.5 cm in length), and cecum were removed, opened lengthwise, and gently flushed with 0.1 M phosphate-buffered salines (pH 7.4) then immediately preserved in 10% neutral buffered formalin solution. To investigate the histological structure, 6–7 thick sections were stained with Delafield’s iron haematoxylin and eosin according to Bancroft and Gamble (2008).

### Histomorphometric investigation of the intestine, spleen, and bursa of Fabricius

Both the depth of the intestinal gland and the villus length, excluding the intestinal crypt, were measured for histomorphometry.

The diameters of the cortical and medullary regions of the follicle of the spleen and bursa of Fabricius were measured (no.). At the Faculty of Veterinary Medicine, Cairo University, stained sections of each bird were evaluated using a Leica Quin 500 analyzer computer system (Leica Microsystems, Switzerland). To transform the measurement units (pixels) generated by the image analyzer program into real micrometer units, the image analyzer was automatically calibrated. For each sample, the photos of each segment were taken at a final magnification of 40 × .

### Transcriptional expression of growth, stress, DNA repair, and immune response-related genes in tissue samples

Real-time PCR (qRT-PCR) was used to determine the expression of *mTOR* (growth-related gene) in breast and thigh muscles, as well as *NBN* (stress and DNA repair-related gene) and *TLR4* (immune response-related gene) in the liver in the broiler chicken (8 birds/group). Total RNA was extracted from the different tissue samples (eight samples of each tissue per group) by an easy-spin Total RNA Extraction Kit (iNtRON Biotechnology DR, Cat. No.17221). The concentration and purity of RNA were evaluated using a Nanodrop ND-1000 spectrophotometer (Nanodrop Technologies) (Kasas et al., 2022). The cDNA synthesis was done by M-MuLV Reverse Transcriptase (NEB#M0253) and the expression levels of mRNA were determined by qRT-PCR using HERA^PLUS^ SYBR Green qPCR kit (#: WF10308002). The using primers were designed by the Primer 3 program (https://primer3.ut.ee) and their sequences are presented in Table 2. (Hassan et al., 2022). Each RT-PCR was carried out 3 times (Kamel et al., 2018). The β-actin gene was used as the reference gene. The obtained data were analyzed using CT, ΔCT, ΔΔCT, and 2^− ΔΔCT^ (Livak and Schmittgen, 2001).

**Table 2:** Primers sequences employed for qRT-PCR

Genes	Gene name	Accession number	Sequence of primers
mTOR	Mechanistic target of rapamycin	XM_417614.6	F: 5′-CGCAGTGAAGAAACAAGGGC-3′ R: 5′-GGTGGCGTTACCTCCTTCAA-3′
NBN	Nibrin	NM_204337.1	F: 5′-GCTTGGAAGGGAAAGTGGTG-3′ R: 5′-TCCCAGTCTAGGTCTCTGCT-3′
TLR-4	Toll-like receptor 4	NM_001030693.1	F: 5′-ATGTCCTCTTGCCATCCCAA-3′ R: 5′-TCTCCCCTTTCTGCAGAGTG-3′
β-actin	Beta-actin	L08165.1	F: 5′-CCCACACCCCTGTGATGAAA-3′ R: 5′-TAGAACTTTGGGGGCGTTCG-3′

### Statistical analysis

PASW Statistics, version 18.0 (SPSS Inc., Chicago, IL, USA) was used for statistical analysis. Results were reported as means and standard errors of means (SEM). One-way ANOVA was applied for comparing the mean values of control and test groups, while post hoc comparisons were determined using Tukey’s test. The threshold of *P* ≤ 0.05 was set for statistical significance. Boxplots were produced by R (R Foundation for Statistical Computing), version 3.6.1 (Team, 2019), based on ggplot2 (Wickham, 2016; Kassambara, 2019) packages.

## Results

### Performance parameters

Table 3 displays the weekly performance parameters of the birds. From day 21 onward, significant increases in body weights were observed in T2 and T3 birds (*P* < 0.05). On day 35, the highest values of body weight gain were recorded for T2 (+ 100 g/bird; + 14.7%), then T3 (+ 86 g/bird; + 12.6%), compared to the control (T1). At the end of the finisher phase (5th week), T2 displayed the lowest average feed consumption, with − 45 g feed/bird (− 3.6%), which was lower than the control (T1) and − 75 g feed/bird (− 5.8%) compared to T3 (*P* < 0.001). On average, the FCR in the 5th week recorded 1.56 g/g (− 16.6%) for T2 and 1.68 g/g (− 10.2%) for T3 compared to 1.87 g/g for the control, with a statistically significant difference of (*P* = 0.012). T2 exhibited the most efficient performance parameters at the grower and finisher phases.

**Table 3:** Influence of dietary *Bacillus* strains probiotics supplements on weekly performance parameters of broiler chickens

Groups*	Body weight (g)					Body weight gain (g)					Feed intake (g)					FCR (g/g)				
	D 7	D 14	D 21	D 28	D 35	D 7	D 14	D 21	D 28	D 35	D 7	D 14	D 21	D 28	D 35	D 7	D 14	D 21	D 28	D 35
T1	192	535	951^b^	1459^b^	2141^b^	148	343	416	508	682^b^	157	419	752	831	1253^a^	1.07	1.22	1.81	1.64	1.87^a^
T2	193	540	980^a^	1507^a^	2289^a^	149	347	443	524	782^a^	158	426	750	824	1208^b^	1.07	1.23	1.70	1.59	1.56^b^
T3	200	549	980^a^	1499^ab^	2268^a^	155	350	430	520	768^ab^	159	420	754	826	1283^a^	1.02	1.20	1.76	1.59	1.68^ab^
SEM^1^	1.90	2.61	5.51	7.46	19.45	1.90	1.82	5.80	6.37	18.56	0.59	2.44	4.71	2.60	8.25	0.02	0.01	0.03	0.02	0.05
*P* value	NS^2^	NS	0.042	0.016	0.001	NS	NS	NS	NS	0.05	NS	NS	NS	NS	0.0001	NS	NS	NS	NS	0.012

^a^^,^^b^Means in the same column showing different superscripts point to significant differences (*P* ≤ 0.05)

T1, Control group (basal diet); T2, basal diet + 0.2 kg multi strains probiotic (*Bacillus coagulans* (DSM 32,806; 2 × 10^9^ cfu/g) and *Bacillus licheniformis* (DSM 33,806; 8 × 10^9^ cfu/g))/ton of feed; T3, basal diet + 0.5 kg single strain probiotic (*Bacillus licheniformis* (DSM 17,236; 3.2 × 10^9^ cfu/g))/ton of feed

*FCR*, feed conversion ratio (g of feed/g of weight gain)

^*^n = 20 birds/group

^1^*SEM* pooled standard error of the mean

^2^*NS* not significant

Table 4 presents the cumulative performance along the 35 days. T2 and T3 showed significantly better body weights, body weight gains, and FCR (*P* = 0.001, 0.001, and 0.0001 respectively) than the control (T1). Feed intake was more efficient in T2 than in T1 and T3 (− 45 and − 75 g feed/bird, respectively) (*P* = 0.014). No significant differences in survival and mortality rates were recorded between treatments (*P* > 0.05). Accordingly, the calculated EPEF revealed a significantly elevated value for T2 when compared to the control (*P* = 0.005).

**Table 4:** Influence of dietary *Bacillus* strains probiotics supplements on cumulative performance parameters of broiler chickens (1–35 days)

Groups	Body weight (g)	Body weight gain (g)	Feed intake (g)	FCR (g/g)	EPEF	Mortality (%)
T1	2141^b^	2097^b^	3413^ab^	1.63^a^	358^b^	4.57
T2	2289^a^	2244^a^	3368^b^	1.50^b^	408^a^	5.50
T3	2268^a^	2223^a^	3443^a^	1.55^b^	391^ab^	6.50
SEM^1^	19.45	19.45	11.10	0.02	6.88	0.60
*P* value	0.001	0.001	0.014	0.0001	0.005	NS^2^

^a^^,^^b^Means in the same column showing different superscript point to significant differences (*P* ≤ 0.05)

T1, Control group (basal diet); T2, basal diet + 0.2 kg multi strains probiotic (*Bacillus coagulans* (DSM 32,806; 2 × 10^9^ cfu/g) and *Bacillus licheniformis* (DSM 33,806; 8 × 10^9^ cfu/g))/ton of feed; T3, basal diet + 0.5 kg single strain probiotic (*Bacillus licheniformis* (DSM 17,236; 3.2 × 10^9^ cfu/g))/ton of feed

*FCR* feed conversion ratio (g of feed/g of weight gain)

*EPEF* European Production Efficiency Factor = (livability × live body weight (kg)/(age in days × FCR) × 100

^1^*SEM* pooled standard error of the mean

^2^*NS* not significant

### Carcass traits and immune organs’ relative weights

In Table 5, T2 and T3 showed an 8.5% increase in the breast weight percentage compared to the control. The bursa weight percentage recorded 26% and 14% increases in T2 and T3, respectively, compared to the control. However, no significant differences in carcass traits (dressing yield, breast, thigh, drum, liver, and gizzard relative weights) or immune organs’ weight percentages were revealed between treatments and control (*P* > 0.05).

**Table 5:** Influence of dietary *Bacillus* strains probiotics supplements on carcass traits of broiler chickens (day 35)

Groups*	Dressing (%)	Breast (%)	Thigh (%)	Drum (%)	Gizzard (%)	Heart (%)	Liver (%)	Bursa (%)	Spleen (%)
T1	75.52	31.61	25.68	13.41	2.35	0.89	3.84	0.27	0.27
T2	72.40	34.38	25.58	13.55	2.63	0.95	3.59	0.34	0.24
T3	71.77	34.22	25.53	13.54	2.73	0.93	3.46	0.31	0.25
SEM^1^	0.77	0.56	0.29	0.18	0.07	0.04	0.10	0.01	0.01
*P* value	NS^2^	NS	NS	NS	NS	NS	NS	NS	NS

^a^^,^^b^Means in the same column showing different superscript point to significant differences (*P* ≤ 0.05)

T1, Control group (basal diet); T2, basal diet + 0.2 kg multi strains probiotic (*Bacillus coagulans* (DSM 32,806; 2 × 10^9^ cfu/g) and *Bacillus licheniformis* (DSM 33,806; 8 × 10^9^ cfu/g))/ton of feed; T3, basal diet + 0.5 kg single strain probiotic (*Bacillus licheniformis* (DSM 17,236; 3.2 × 10^9^ cfu/g))/ton of feed

^*^n = 8 birds/group

^1^*SEM* pooled standard error of the mean

^2^*NS* not significant

### Bacterial counts of cloacal swabs and cecal content

**Fig.1:**
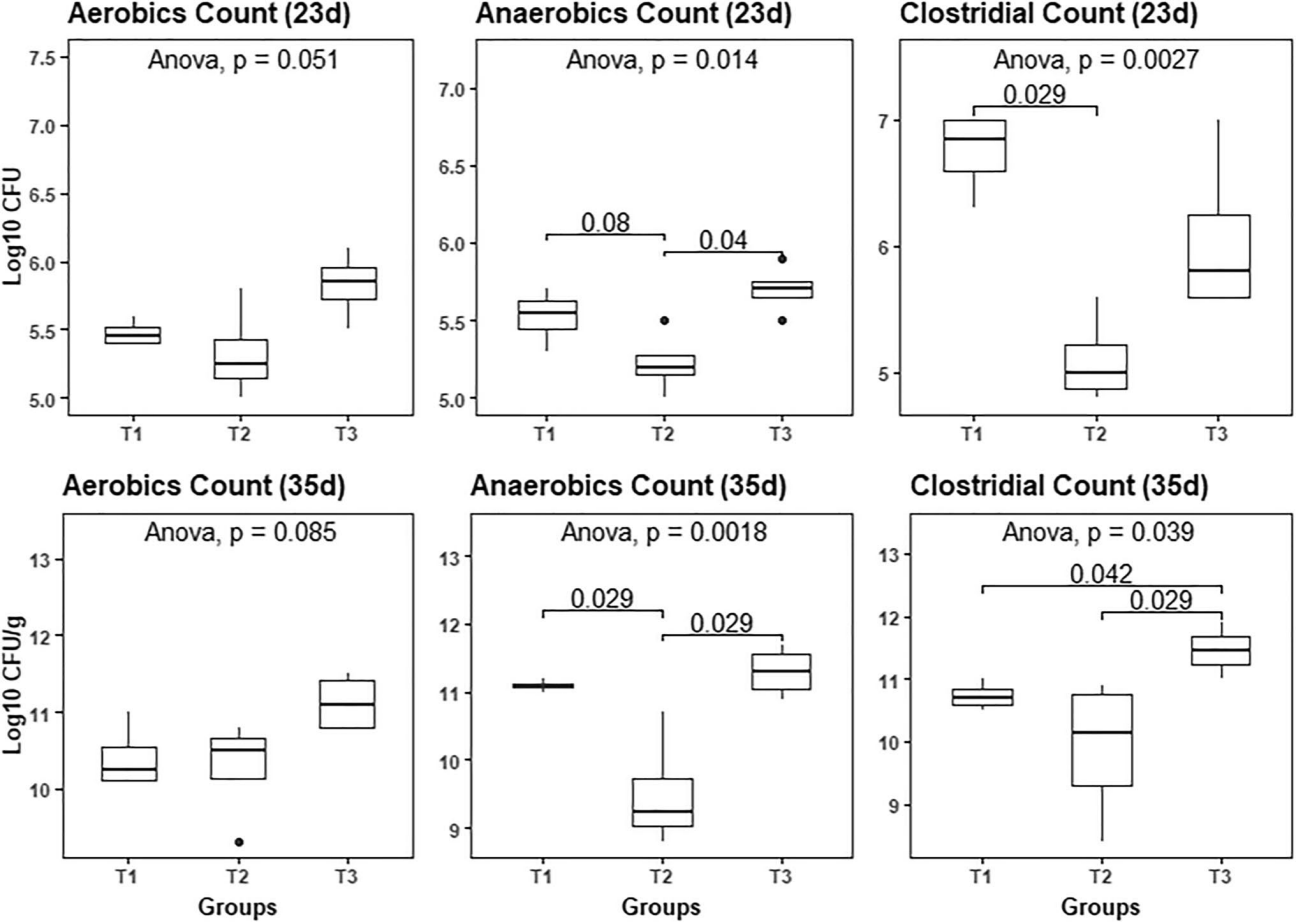
Influence of dietary *Bacillus* strains probiotic supplements on cloacal (day 23) and cecal (day 35) bacterial counts of broiler chickens (*n* = 8 birds/group); T1, control group (basal diet); T2, basal diet + 0.2 kg multi strains probiotic (*Bacillus coagulans* (DSM 32,806; 2 × 10^9^ cfu/g) and *Bacillus licheniformis* (DSM 33,806; 8 × 10^9^ cfu/g))/ton of feed; T3, basal diet + 0.5 kg single strain probiotic (*Bacillus licheniformis* (DSM 17,236; 3.2 × 10.^9^ cfu/g))/ton of feed; significance was indicated at *P* ≤ 0.05

Figure 1 presents the aerobic, anaerobic, and clostridial bacterial counts of cloacal swabs (day 23) and cecal contents (day 35). From day 23 to day 35, the overall bacterial counts exhibited a 4.72–5.15 log_10_ CFU increase. On day 23, cloacal swabs of T2 recorded the lowest aerobic, anaerobic, and clostridial bacterial counts (means = 5.33, 5.23, and 5.10 log_10_ CFU, respectively), with significant differences with the control (T1) (means = 5.48, 5.53, and 6.75 log_10_ CFU, respectively) and T3 (means = 5.83, 5.70, and 6.05 log_10_ CFU, respectively) (*P* = 0.014). On day 35, T2 birds displayed the lowest cecal aerobic, anaerobic, and clostridial bacterial counts (means = 10.28, 9.50, and 9.90 log_10_ CFU/g, respectively), and these values differed significantly from those of the control (means = 10.40, 11.10, and 10.73 log_10_ CFU/g, respectively), and T3 (means = 11.13, 11.30, and 11.45 log_10_ CFU/g, respectively). Unfortunately, T3 did not show efficiency in lowering cecal microbial counts (especially clostridial counts) at any age compared to the control (T1).

### Litter chemical and microbiological qualities

Table 6 presents data on litter quality. On day 35, litter nitrogen was significantly reduced in T2 and T3 compared to the control T1, with T2 recording the lowest significance level (*P* = 0.0001). On the other hand, the peak of litter moisture was recorded on day 23 in all groups after feeding the birds the mash grower diet. On day 35, T2 showed the lowest litter moisture while T3 showed the highest level; however, the differences were not statistically significant (*P* > 0.05). Bacteriological examination of litter revealed relatively higher bacterial counts in T1 compared to T2 and T3. However, T3 showed elevated litter bacterial counts on day 35, primarily for *Clostridia*.

**Table 6:** Influence of dietary *Bacillus* strains probiotics supplements on the chemical and microbiological qualities of built-up litter of broiler chicken

Groups*	Nitrogen (g/kg)	Moisture (g/kg)			Total aerobic count (mean log_10_ CFU/g)			Total anaerobic count (mean log_10_ CFU/g)			Clostridial count (mean log_10_ CFU/g)		
	D 35	D 10	D 23	D 35	D 10	D 23	D 35	D 10	D 23	D 35	D 10	D 23	D 35
T1	37.22^a^	278	468	345	7.17^a^	9.93	13.40	6.65	9.74	13.10	7.09	9.44	13.50
T2	23.81^c^	334	517	318	6.34^b^	9.75	13.48	6.87	9.64	13.03	6.73	9.38	13.43
T3	25.23^b^	307	424	393	6.30^b^	9.61	13.75	6.26	9.28	13.35	6.55	9.48	13.70
SEM^1^	2.13	19.80	19.24	16.50	0.15	0.08	0.09	0.24	0.10	0.09	0.19	0.07	0.07
*P* value	0.0001	NS^2^	NS	NS	0.008	NS	NS	NS	NS	NS	NS	NS	NS

^a^^,^^b,c^Means in the same column showing different superscript point to significant differences (*P* ≤ 0.05)

T1, Control group (basal diet); T2, basal diet + 0.2 kg multi strains probiotic (*Bacillus coagulans* (DSM 32,806; 2 × 10^9^ cfu/g) and *Bacillus licheniformis* (DSM 33,806; 8 × 10^9^ cfu/g))/ton of feed; T3, basal diet + 0.5 kg single strain probiotic (*Bacillus licheniformis* (DSM 17,236; 3.2 × 10^9^ cfu/g))/ton of feed

^*^n = 8 samples/group

^1^*SEM* pooled standard error of the mean

^2^*NS* not significant

### Confirmation and toxin typing of *Clostridium perfringens*

The multiplex PCR of suspected *C. perfringens* isolates revealed that the amplification of the alpha-toxin gene at 324 bp represented *C. perfringens* type A.

### Blood biochemical parameters

Serum biochemistry results are presented in Table 7. T2 showed an 11% increase in serum albumin and a 6.3% reduction in uric acid compared to the control. However, no significant differences were found between the groups in the uric acid or protein profiles of sera (total protein, albumin, globulin, and A\G ratio). The cholesterol concentration significantly decreased in T3 (*P* = 0.001). AST, ALT, LDH, and ALP activities were significantly reduced in T2 and T3 compared to T1 (*P* < 0.05).

**Table 7:** Influence of dietary *Bacillus* strains probiotics supplements on blood biochemical parameters of broiler chickens (day 35)

Groups*	Protein profile				Lipid profile		Liver and other organs functions’ parameters				Kidney function
	Total protein (g\dl)	Albumin (g\dl)	Globulin (g\dl)	A\G	Cholesterol (mg\dl)	TAG (mg\dl)	ALT (U\L)	AST (U\L)	LDH (U\L)	ALP (U\L)	Uric acid (mg\dl)
T1	2.20	1.47	0.63	2.37	133.04^a^	69.76	21.25^a^	224.65^a^	2251.31^a^	2002.75^a^	2.22
T2	2.08	1.63	0.53	3.10	116.08^a^	53.19	11.45^b^	149.71^b^	1403.13^b^	1508.33^ab^	2.08
T3	2.21	1.54	0.67	2.44	76.66^b^	69.38	11.64^b^	176.06^b^	1739.53^ab^	1325.32^b^	2.58
SEM^1^	0.059	0.067	0.041	0.222	8.13	3.89	1.66	11.67	144.01	120.15	0.19
*P* value	NS^2^	NS	NS	NS	0.001	NS	< 0.0001	0.002	0.019	0.050	NS

^a^^,^^b^Means in the same column showing different superscript point to significant differences (*P* ≤ 0.05)

T1, Control group (basal diet); T2, basal diet + 0.2 kg multi strains probiotic (*Bacillus coagulans* (DSM 32,806; 2 × 10^9^ cfu/g) and *Bacillus licheniformis* (DSM 33,806; 8 × 10^9^ cfu/g))/ton of feed; T3, basal diet + 0.5 kg single strain probiotic (*Bacillus licheniformis* (DSM 17,236; 3.2 × 10^9^ cfu/g))/ton of feed

*A\G*, albumin\globulin ratio; *TAG*, triacylglyceride; *ALT*, Alanine transaminase; *AST*, aspartate transaminase; *LDH*, lactate dehydrogenase; *ALP*, alkaline phosphatase

^*^n = 8 birds/group

^1^*SEM* pooled standard error of the mean

^2^*NS* not significant

### ND vaccinal HI titers

**Fig. 2:**
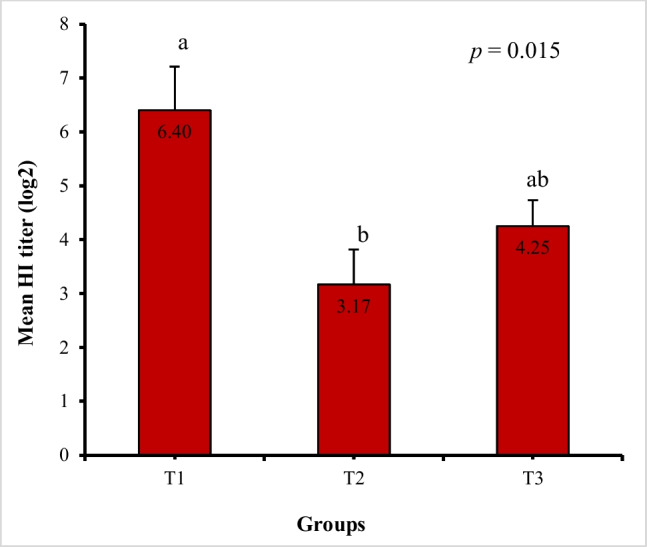
Influence of dietary *Bacillus* strains probiotic supplements on serological mean HI titers (log_2_) toward NDV vaccination of broiler chickens (*n = 8 birds/group). Data are represented as mean ± SEM. ^a,b^ Bars showing different superscripts point to significant differences (*P* ≤ 0.05). T1, control group (basal diet); T2, basal diet + 0.2 kg multi strains probiotic (*Bacillus coagulans* (DSM 32,806; 2 × 10^9^ cfu/g) and *Bacillus licheniformis* (DSM 33,806; 8 × 10^9^ cfu/g))/ton of feed; T3, basal diet + 0.5 kg single strain probiotic (*Bacillus licheniformis* (DSM 17,236; 3.2 × 10^9^ cfu/g))/ton of feed

The results of HI titers against NDV are displayed in Fig. 2. T2 and T3 exhibited lower HI titers (3.17 ± 0.65 and 4.25 ± 0.48 log_2_, respectively) compared to T1 (6.40 ± 0.81 log_2_); (*P* = 0.015).

### Intestinal and immune organ histology and histomorphometry

#### Histology of the liver, small intestine, and large intestine

**Fig. 3:**
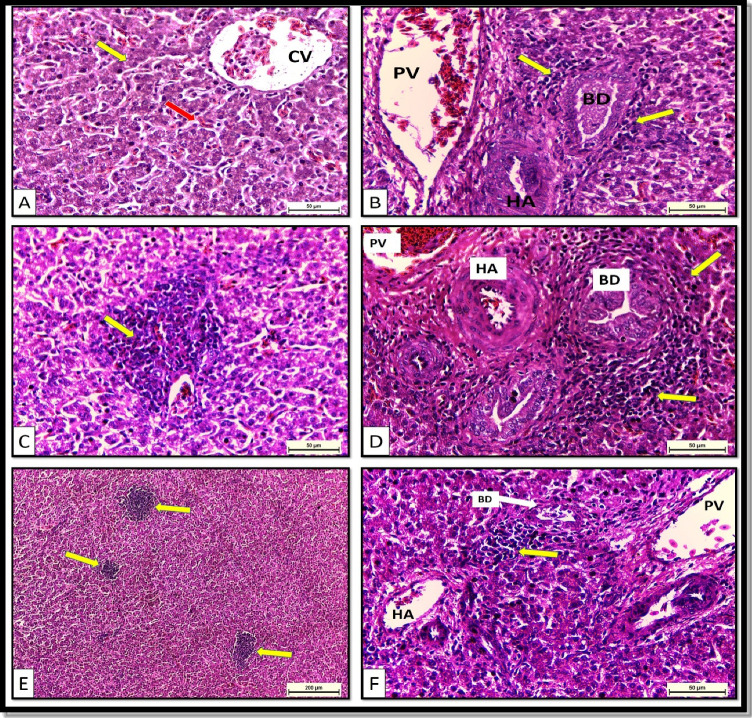
Liver sections of chickens. (**A–C**) Control group (T1) demonstrating **A** normal hepatic parenchyma, hepatic cord (yellow arrow) radiating from the central vein (CV), and blood sinusoids (red arrow). H&E. X400 **B** Portal area containing bile duct (BD), hepatic artery (HA), and portal vein (PV) with normal lymphocytic infiltration (arrows). H&E. X400 **C** Lymphocytic and eosinophilic leukocytic aggregations (arrow) around the vessels and between the cords. H&E. X400. (**D**, **E**) T2, basal diet + 0.2 kg multi strains probiotic (*Bacillus coagulans* (DSM 32,806; 2 × 10^9^ cfu/g) and *Bacillus licheniformis* (DSM 33,806; 8 × 10^9^ cfu/g))/ton of feed showing (**D**) sever increase in the lymphocytic infiltration (arrows) within the portal area. Notice: bile duct (BD), hepatic artery (HA), and portal vein (PV). H&E. X400. **E** Increase lymphocytic and eosinophilic leukocytic aggregations (arrows) around the vessels and between the cords. H&E. X100. **F** T3, basal diet + 0.5 kg single strain probiotic (*Bacillus licheniformis* (DSM 17,236; 3.2 × 10.^9^ cfu/g))/ton of feed showing mild increase in the lymphocytic infiltration (arrow) within the portal area. Notice: bile duct (BD), hepatic artery (HA), and portal vein (PV). H&E. X400

The histological findings of the liver are presented in Fig. 3. The histological structure of the liver parenchyma was normal in T1, as the hepatocytes were radiated from the central vein in cords in between the blood sinusoids (Fig. 3A). The portal area contained the bile duct, hepatic artery, and portal vein with normal lymphocytic infiltration (Fig. 3B). Lymphocytic and eosinophilic leukocytic aggregations were normally found around the vessels and between the cords (Fig. 3C). T2 showed a severe increase in the lymphocytic infiltration within the portal area compared with the control (Fig. 3D). Moreover, the lymphocytic and eosinophilic leukocytic aggregations were increased around the vessels and between the cords (Fig. 3E). Additionally, a mild increase in the lymphocytic infiltration was observed within the portal area in liver tissues of T3 compared with those of T2 (Fig. 3F).

**Fig. 4:**
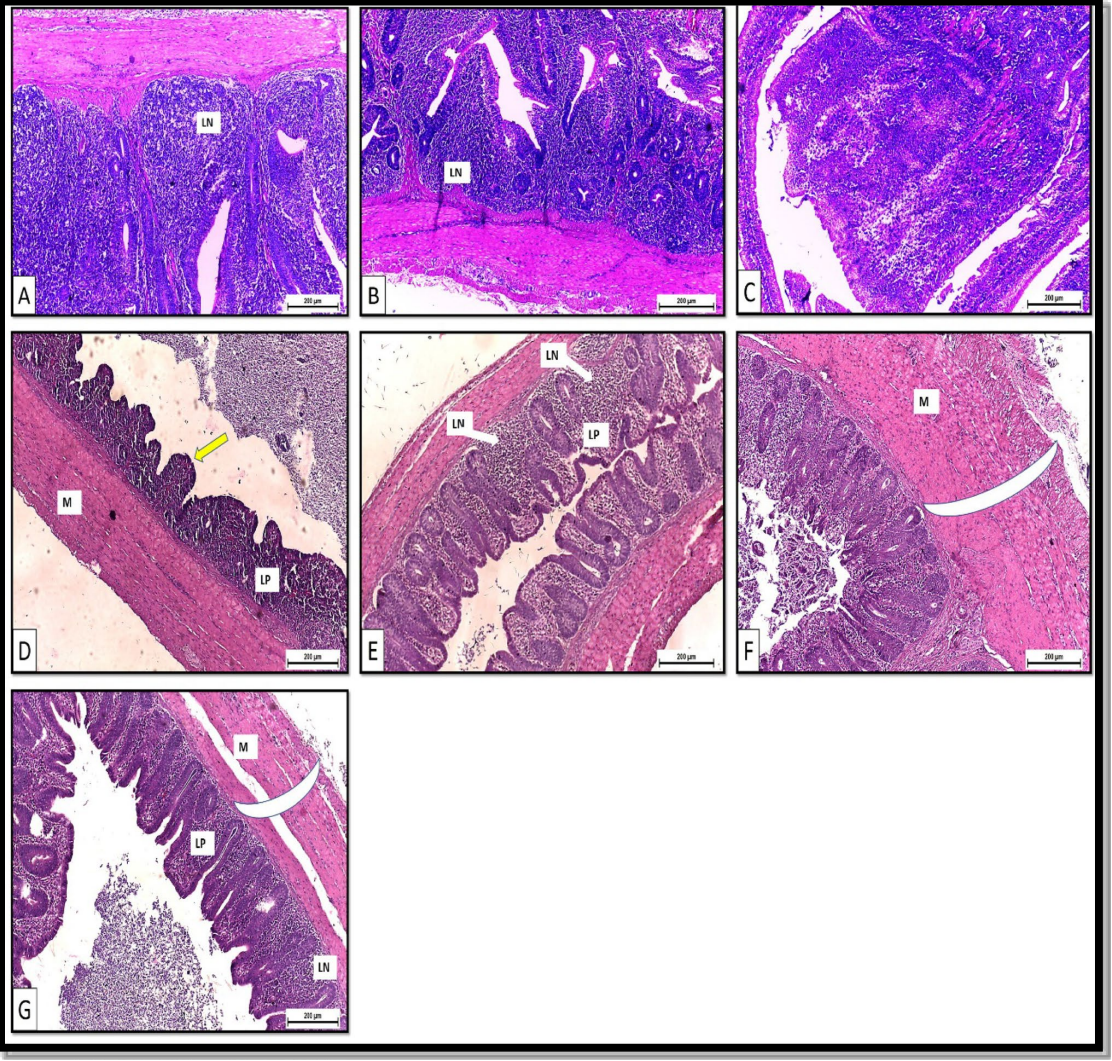
(**A–C**) Small intestine sections from poultry. **A**, **B** T2, basal diet + 0.2 kg multi strains probiotic (*Bacillus coagulans* (DSM 32,806; 2 × 10^9^ cfu/g) and *Bacillus licheniformis* (DSM 33,806; 8 × 10^9^ cfu/g))/ton of feed showing appearance of lymph nodules (LN) in lamina propria. H&E. X100 **A** Duodenum **B** jejunum **C** T3, basal diet + 0.5 kg single strain probiotic (*Bacillus licheniformis* (DSM 17,236; 3.2 × 10^9^ cfu/g))/ton of feed group (T3) showing duodenum with increase in the lymphoid tissue of the lamina propria. H&E. X100. (**D–G**) Large intestine sections from poultry **(D)** control group (T1) showing short intestinal villi (arrow), lamina propria (LP) containing lymphoid cells and lymph nodules, and normal thickness of tunica muscularis (M). H&E. X100. **E**, **F** T2, basal diet + 0.2 kg multi strains probiotic (*Bacillus coagulans* (DSM 32,806; 2 × 10^9^ cfu/g) and *Bacillus licheniformis* (DSM 33,806; 8 × 10^9^ cfu/g))/ton of feed showing (**E**) sever increase in the lymphoid tissue and lymph nodules (LN) of the lamina propria (LP). H&E. X100. **F** Increase in the thickness (arc) of the tunica muscularis (M). H&E. X100. **G** T3, basal diet + 0.5 kg single strain probiotic (*Bacillus licheniformis* (DSM 17,236; 3.2 × 10.^9^ cfu/g))/ton of feed showing mild increase in the lymphoid tissue and lymph nodules (LN) of the lamina propria (LP) and in the thickness (arc) of the tunica muscularis (M). H&E. X100

The histological results of the sections of the small intestines are presented in Fig. 4. T2 showed the appearance of lymph nodules in the lamina propria of the duodenum and jejunum (Fig. 4A and B). Meanwhile, T3 showed an increase in the lymphoid tissue of the lamina propria of the duodenum only (Fig. 4C). Examination of large intestine sections of the control (T1) revealed a normal histological structure with short intestinal villi. The lamina propria contained lymphoid cells and lymph nodules with a normal thickness of the tunica muscularis (Fig. 4D). However, large intestine sections from birds of T2 showed a severe increase in the lymphoid tissue and lymph nodules of the lamina propria (Fig. 4E). Moreover, a noticeable increase in the thickness of the tunica muscularis was observed compared with the control (Fig. 4F). A mild increase in the lymphoid tissue and lymph nodules of the lamina propria and the thickness of the tunica muscularis were also noticed within the large intestine tissues of T3 compared with T2 (Fig. 4G).

#### Histomorphometry of the small intestine, spleen, and bursa

The results in Table 8 present the histomorphometry of the small intestine. The length of the intestinal villi of the duodenum, jejunum, and ilium showed significant increases in T2 and T3 compared to T1. However, T2 and T3 had significantly reduced duodenal crypt depths compared to T1. Deeper crypts were recorded in T2’s jejunum and T3’s ileum (*P* = 0.0001).

**Table 8:** Influence of dietary *Bacillus* strains probiotics supplements on histomorphometry of small intestine and immune organs of broiler chickens (mean µm)

Groups*	Duodenum		Jejunum		Ileum	
	Villi length	Crypt depth	Villi length	Crypt depth	Villi length	Crypt depth
T1	1257^b^	144^a^	1027^b^	113^b^	752^c^	115^b^
T2	1677^a^	116^b^	1250^a^	140^a^	810^b^	121^ab^
T3	1802^a^	100^c^	1333^a^	115^b^	1031^a^	126^a^
SEM^1^	74.027	5.904	55.491	5.098	17.530	3.841
*P* value	0.0001	0.0001	0.0001	0.0001	0.0001	0.0001

^a^^,^^b,c^Means in the same column showing different superscript point to significant differences (*P* ≤ 0.05)

T1, Control group (basal diet); T2, basal diet + 0.2 kg multi strains probiotic (*Bacillus coagulans* (DSM 32,806; 2 × 10^9^ cfu/g) and *Bacillus licheniformis* (DSM 33,806; 8 × 10^9^ cfu/g))/ton of feed; T3, basal diet + 0.5 kg single strain probiotic (*Bacillus licheniformis* (DSM 17,236; 3.2 × 10^9^ cfu/g))/ton of feed

^*^n = 8 birds/group

^1^*SEM* pooled standard error of the mean

**Fig. 5:**
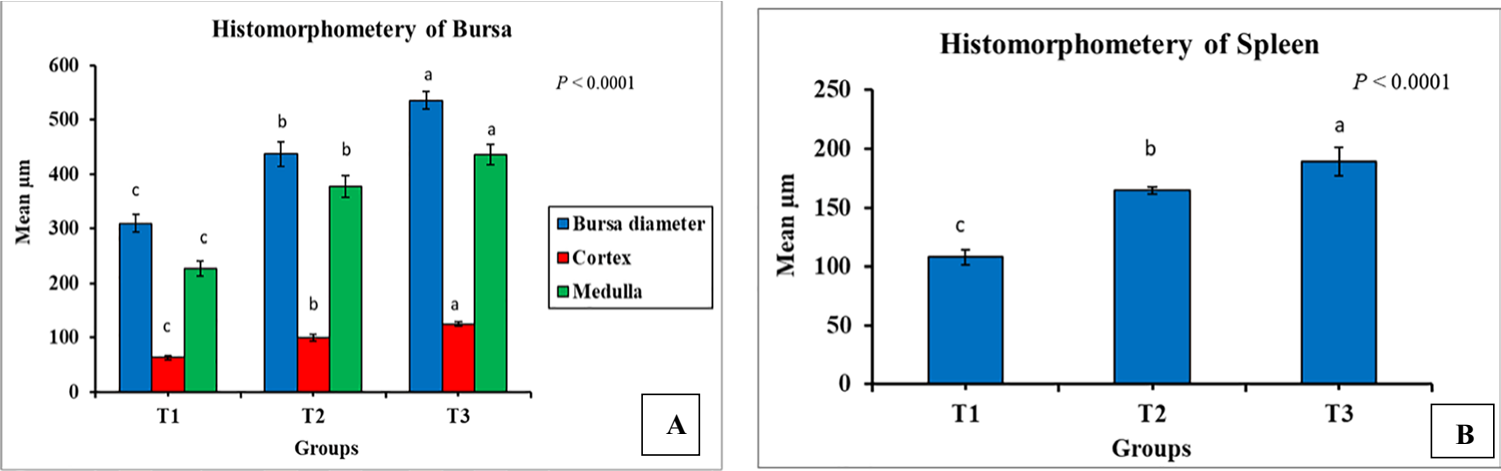
Influence of dietary *Bacillus* strains probiotics supplements on histomorphometry immune organs of broiler chickens (*n* = 8 birds/group). **A** Bursa and **B** spleen. Data are represented as mean µm and SEM. ^a,b,c^ Bars showing different superscripts point to significant differences (*P* ≤ 0.05). T1, control group (basal diet); T2, basal diet + 0.2 kg multi strains probiotic (*Bacillus coagulans* (DSM 32,806; 2 × 10^9^ cfu/g) and *Bacillus licheniformis* (DSM 33,806; 8 × 10^9^ cfu/g))/ton of feed; T3, basal diet + 0.5 kg single strain probiotic (*Bacillus licheniformis* (DSM 17,236; 3.2 × 10^9^ cfu/g))/ton of feed

The results in Fig. 5 introduced the histomorphometry of immune organs (spleen and bursa). T3 and T2 showed significantly higher diameters of the bursa (including the cortex and medulla) and the white pulp of the spleen (*P* < 0.0001) than those of the control. Eventually, T3 recorded the maximum immune organ measures compared to T2 and T1.

### Gene expression

**Fig. 6:**
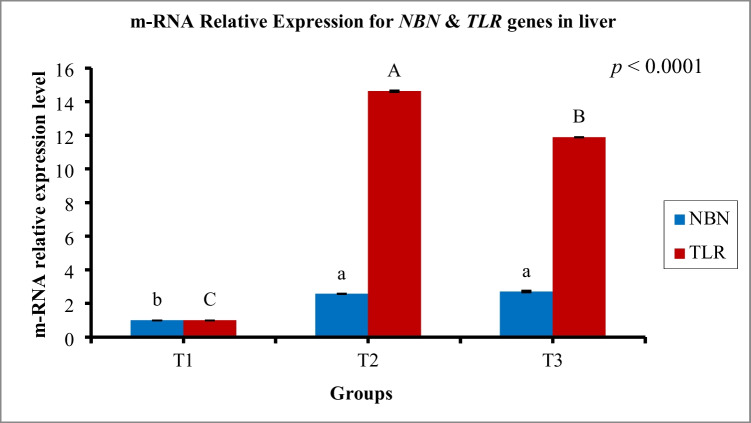
Influence of dietary *Bacillus* strains probiotics supplements on m-RNA relative expression levels of the different genes; *NBN* (stress and DNA repair-related gene) and *TLR* (immune response-related gene) in the liver tissue of broiler chickens (*n* = 8 birds/group). Data are represented as means and SEM. ^a,b,c^ and ^A,B,C^ Bars showing different superscripts point to significant differences (*P* ≤ 0.05). T1, control group (basal diet); T2, basal diet + 0.2 kg multi strains probiotic (*Bacillus coagulans* (DSM 32,806; 2 × 10^9^ cfu/g) and *Bacillus licheniformis* (DSM 33,806; 8 × 10^9^ cfu/g))/ton of feed; T3, basal diet + 0.5 kg single strain probiotic (*Bacillus licheniformis* (DSM 17,236; 3.2 × 10^9^ cfu/g))/ton of feed

**Fig. 7:**
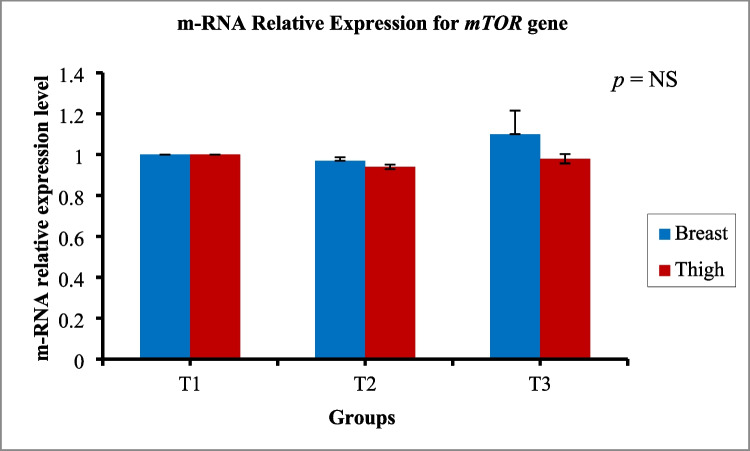
Influence of dietary supplementation of *Bacillus* strains probiotics on m-RNA relative expression levels for *mTOR* gene (growth-related gene) in both breast and thigh muscles of broiler chickens (*n* = 8 birds/group). Data are represented as means and SEM. Significant difference was set at *P* ≤ 0.05. T1, control group (basal diet); T2, basal diet + 0.2 kg multi strains probiotic (*Bacillus coagulans* (DSM 32,806; 2 × 10^9^ cfu/g) and *Bacillus licheniformis* (DSM 33,806; 8 × 10^9^ cfu/g))/ton of feed; T3, basal diet + 0.5 kg single strain probiotic (*Bacillus licheniformis* (DSM 17,236; 3.2 × 10^9^ cfu/g))/ton of feed

In Figs. 6 and 7, the mRNA relative expression levels of *NBN*, *TLR*, and *mTOR* tissue genes were plotted. In liver tissues of T2 and T3, the transcript levels of *NBN* (stress and DNA repair-related gene) and *TLR* (immune response-related gene) genes were significantly more upregulated than those in T1. The upregulation of *TLR* mRNA was the highest in T2 (Fig. 6). The transcript level of the *mTOR* gene (growth-related gene) was examined in both the breast and thigh muscles of broiler chickens. There were no significant differences in the expression level of the *mTOR* gene between the three groups (Fig. 7).

## Discussion

In this study, we explored the effects of adding *Bacillus* as a probiotic feed supplement to broiler chickens, using either a single strain product (*Bacillus licheniformis*) or a multi-strain formula of *Bacillus coagulans* and *Bacillus licheniformis*. Our results revealed that the two formulations of *Bacillus* probiotics enhanced the weekly and overall growth performance of the birds in T2 and T3, especially from day 21 onward. Bacilli probiotics were thought to express their growth-enhancing impacts at later growth stages (Upadhaya et al., 2019a, b; Ahmat et al., 2021). However, feeding birds the probiotic with two *Bacillus* strains (T2 = *Bacillus coagulans* + *Bacillus licheniformis*) achieved a better growth rate and more efficient feed intake than feeding them a single *Bacillus* probiotic (T3 = *Bacillus licheniformis*). This significant improvement could be due to the growth-promoting effect of *Bacillus coagulans* via the synthesis and metabolism of proteins, short-chain fatty acids, and vitamins (LeBlanc et al., 2017). Once activated and germinated, *Bacillus coagulans* may aid in digesting carbohydrates and proteins, helping the birds gain weight (Maathuis et al., 2010). Moreover, adding multiple strains of *Bacillus* to broiler rations significantly enhanced body weight gain and the FCR (Ahmat et al., 2021; Wealleans et al., 2017).

Although the grower feed was administered as mash instead of pellets, birds supplemented with both *Bacillus coagulans* and *Bacillus licheniformis* (T2) overcame that stressor and achieved an improved FCR. These findings could be attributed to the enhanced secretion of endogenous enzymes (such as amylase, protease, and lipase) stimulated by *Bacillus coagulans*, which boosts feed digestibility and availability, and intestinal peristalsis (Upadhaya et al., 2019a, b; LeBlanc et al., 2017; Gu et al., 2015). Furthermore, *Bacillus coagulans* may improve the feed conversion ratio of broiler chickens by promoting a healthy balance of intestinal microbiota (Hung et al., 2012). Bacilli-supplemented birds in T2 showed higher EPEF values than those in the control group, which indicated the improvement of economic benefits as stated by Zhang et al. (2021).

The results of the current study revealed that dietary probiotics of multiple strains (T2) increased the relative weight (%) of breast muscle compared to the control and T3, and similar findings were reported by Ahmat et al. (2021). Other carcass traits did not exhibit any increase due to the inclusion of *Bacillus* probiotics compared to the control, which was in line with the findings of other previous studies (Upadhaya et al., 2019a, b; Bahrampour et al., 2020; Upadhaya et al., 2019a, b). Dietary supplementation with *B. coagulans and/or B. licheniformis* increased bursa relative weights in T2 and T3 birds. The bursa of Fabricius is responsible for humoral immunity in birds. Bacillus-based probiotics have the ability to regulate broilers’ immune systems (Ahmat et al., 2021). Other studies had reported that bacilli-based probiotics had no effect on the weight of immune organs (spleen and bursa) (Ghahri et al., 2013; Yun et al., 2017). NDV antibody titers in T3 supplemented with 0.5 kg *Bacillus licheniformis* probiotic/ton were higher than in T2 supplemented with 0.2 kg Bacilli probiotic/ton. This variation could be attributed to the different dosages administered to the treatment groups (Ahmat et al., 2021) or strain-to-strain variability.

The results of serum biochemistry revealed that T2 expressed higher serum albumin concentrations than the control, even though the differences were not statistically significant. Zhu et al. (2020) reported that there were no significant differences in the plasma concentrations of total protein, albumin, and triglyceride because of heat-inactivated probiotics. Albumin, which is produced mainly by the liver, is a principal element of total protein. The increased amount of serum albumin in bacilli-supplemented birds was attributed to the antibacterial metabolites secreted by *Bacillus* that inhibit the growth of pathogens and enhance the utilization of dietary protein (Ahmat et al., 2021). Adding multi-*Bacillus* strains to T2 reduced serum uric acid levels compared to the control. Uric acid is the nitrogenous excretory product of protein metabolism in birds and the measurement of its serum levels is one of the renal function tests. This result indicated that bacilli probiotics reduced the pressure on birds’ kidneys by reducing the serum non-protein nitrogen as uric acid (Ahmat et al., 2021).

Serum cholesterol levels in T3 and T2 were lower than in the control group. Cholesterol is included in developing cell membranes, and bile formation, and is a precursor of vitamin D and many hormones. The hypocholesterolemic effect of both probiotics is attributed to their ability to bind cholesterol in the small intestine (Ahmat et al., 2021; Manafi et al., 2017). Serum TAG decreased due to the addition of the combined *Bacillus coagulans* and *Bacillus licheniformis* probiotic compared to the control; however, the decrease was not statistically significant as reported previously (Zhu et al., 2020). Zhou et al. (2016) reported that *B. licheniformis* can improve lipid metabolism in mice that were fed a high-fat diet.

Enhanced liver functions were identified in sera of T2 and T3 as reduced ALT and AST concentrations, which indicated the significant efficacy of bacilli in the protection of treated birds from hepatocellular damage compared to controls (Hussein et al., 2020). Also, bacilli probiotics lowered ALP and LDH activities in T2 and T3 sera. The ALP enzyme is considered a biomarker for other tissues such as bone and renal tissues. The decrease in ALP activity indicates the ameliorative effect of both probiotics on bone and kidney functions (Sharma et al., 2014; Rashidi et al., 2020). Additionally, LDH is recognized as a marker for cell injuries and tissue damage. The obtained results suggested a decline in LDH activity in the serum of broiler chickens that were fed bacilli probiotics indicating fewer cell injuries (Mazkour et al., 2020).

The expressions of the *NBN*, *TLR*4, and *mTOR* genes and their relationship with the body performance of broiler chickens were investigated. The *NBN* gene codes for the Nibrin protein, which is involved in many critical cellular functions, including DNA repair of damaged lesions by guiding repair proteins to sites of DNA damage, which in turn prevents uncontrolled cell division and/or death (Khalaf et al., 2020). T2 and T3 expressed significantly higher levels of *NBN* compared to T1. This could be because the change in the physical form of the grower diet to mash instead of pellets may cause stress on broiler chickens. Stress may increase damage to biomolecules, including DNA (Czarny et al., 2018). Stress-induced DNA damage might stimulate the expression of the *NBN* gene to provide genomic stability and DNA repair which appeared in the groups that were fed *Bacillus* probiotics. Moreover, a significant increase in *TLR*4 gene expression in T2 and T3 was observed. *TLR*s are evolutionarily conserved membrane receptors widely expressed on various innate immunity cells and non-immune cells for sensing abnormal factors, stimulating the innate immune system’s response (Rozs et al., 2001). Stress has been associated with changes in immune parameters, including an increase in innate immunity. *TLR*s appear as the first line of defense against invading environmental stressors, and their expression is modulated (Gárate et al., 2013). Gárate et al. (2013) reported that stress exposure upregulated the *TLR*-4 pathway in the prefrontal cortex of mice. On the other hand, the transcript level of the *mTOR* gene did not differ significantly between the three groups. The *mTOR* gene is responsible for regulating cell growth and metabolism in response to growth factors and nutrients (Saxton and Sabatini 2017). In the current study, an increased immune response, which is an energy-demanding process that diverts nutrients from growth, and subsequently reduces performance, was observed (Emami et al., 2020). This might explain the non-significant differences in carcass traits between the treated groups and the control group.

Histomorphologic investigations of immune organs revealed that T2 and T3 displayed larger diameters of the white pulp of the spleen and follicle of the bursa compared to the control, with T3 recording the largest diameters for both organs. In addition, T2 demonstrated a significant boost of lymphocytic infiltration in the portal area of the liver, while T3 showed a mild increase in lymphocytic infiltration. From these observations, there was a general increase in the immunity induced by *Bacillus* probiotics, which come in agreement with the upregulation of *TLR4* gene expression in the liver of treated groups. Mingmongkolchai and Panbangred (2018) reported that immune function modulations of host defense systems are necessary features of prospective probiotics. According to Duc et al. (2004) and Xu et al. (2012), *Bacillus* spores have been shown to improve innate immunity and macrophage phagocytosis.

Examinations of the small intestines of T2 birds revealed the appearance of lymph nodules in the lamina propria of the duodenum and jejunum, with a severe increase in the large intestine. Meanwhile, the same examinations in T3 birds revealed an increase in the lymphoid tissue of the lamina propria of the duodenum and a mild increase in the large intestine. *Bacillus* surface-associated proteins (such as S-layer proteins, aminopeptidase, flagellin, and cell envelop-bound metalloprotease) are specifically attached to mucin and fibronectin and may play a role in the probiotic strain’s adherence to the GIT (Sánchez et al., 2009). Bacterial adhesion to intestinal epithelial cells not only helps them colonize the gut but can also stimulate immune cells in the gut’s lymphoid tissue. According to Duc et al. (2004), *Bacillus* spores may penetrate microfold (M) cells and migrate through Peyer’s patches before being delivered to efferent lymph nodes. Antigen-presenting cells (dendritic cells and macrophages) are abundant in Peyer’s patches and are important in processing and presenting antigens to B cells for the synthesis of secretory IgA (sIgA), which are required for immunity against mucosal infections.

According to Sen et al. (2012), *Bacillus* generates micromorphological changes in the gut of broilers, increasing the villus height, as well as the villus-height-to-crypt-depth ratio, hence improving the small intestine’s nutritional digestibility and absorption capacity. This finding was in line with the significant difference we found in the length of the intestinal villi of the duodenum, jejunum, and ilium in T2 and T3 when compared to T1. Moreover, there was a significant difference in the crypt depth of the duodenum in T2 and T3 when compared to T1.

From the microbiological perspective, our results revealed the variable effects of *Bacillus* supplements on intestinal microbial counts during different stages of age. T2 showed the lowest counts of aerobic, anaerobic, and clostridial bacteria. There was no difference in intestinal microbial counts between T3 and the control (T1). These results indicated that there were significant reductions in gut bacterial counts, especially for *Clostridia*, in the multi-bacilli probiotic-treated group (T2). Several studies evaluated the effects of *Bacillus* on enhancing gut microflora and indicated that *Bacillus* produces peptides with powerful antimicrobial efficacy that suppress the multiplication of pathogenic bacteria like *Clostridium perfringens* and prevent the development of necrotic enteritis in broilers (Whelan et al., 2019; Sandvang et al., 2021). According to Sandvang et al. (2021), combining multiple strains of bacilli allowed the complementary interaction of the antibacterial effects of each single *Bacillus* strain. The results of the litter microbiological examination revealed that T2 and T3 displayed variable lower litter bacterial counts than the control (T1). These findings are in line with those of De Cesare et al. (2019), who reported that *Bacillus* spores showed the ability to inhabit the broilers’ litter and subsequently reduce the mean bacterial counts.

Physical examination of deep litter revealed that T2 showed the lowest level of litter moisture at the end of the experiment (day 35) but T3 showed high levels of litter moisture. High litter moisture activates bacterial growth and stimulates the anaerobic fermentation of litter (Payne et al., 2007; Sharma et al., 2017). These results are in line with the findings of Ribeiro et al. (2014) who reported that adding a high concentration of *Bacillus* to birds’ diets improved the moisture content of their excreta.

The nitrogen content of deep litter reported the lowest litter nitrogen levels in T2, followed by T3, which indicated adequate nitrogen digestion and absorption inside the intestinal tracts of birds. These results indicated the positive effect of supplementing broiler feed with *Bacillus* to enhance protein digestion and absorption and enhance the quality of the litter. Shojadoost et al. (2012) and Latorre et al. (2015) concluded that *Bacillus* feed supplements improved protein digestion and, subsequently, reduced the protein available to bacterial growth. Additionally, as litter nitrogen content drops, ammonia emissions in the broiler house are reduced and ammonia pollution of the environment is decreased. Ammonia, which is a significant air pollutant produced mainly from intensive animal and poultry production systems, possesses health hazards for animals and humans (Wang et al., 2009). Ferket et al. (2002) reported findings that were in line with these ones, as they indicated that ammonia emission is correlated to the intestinal microbiome and the utilization of nutrients. Jeong and Kim (2014) reported that broiler feed supplementation with *Bacillus* significantly reduced ammonia emissions from litter.

## Conclusion

The long-term usage of antibiotic growth promoters (AGP) may induce bacterial resistance to antibiotics and raise antibiotic residual levels in animal products, which threatens both veterinary and public health. Probiotics are non-pathogenic microorganisms that are potentially important non-antibiotic alternatives. The present study concluded that the novel multi-strain *Bacillus*-based probiotic has a higher positive impact in promoting production profitability by encouraging bird growth and improving feed intake and FCR than a single-strain *Bacillus*-based probiotic. improving intestinal morphology and immune organs, upregulating immunity-related genes, all these inhibit harmful intestinal bacteria and sustain litter quality.

## Data Availability

All data generated or analyzed during this study are included in this published article.
